# Response to treatment of hepatitis C in HCV/HIV co-infected patients is not influenced by either abacavir or tenofovir with weight-based ribavirin

**DOI:** 10.1186/1758-2652-13-S4-P211

**Published:** 2010-11-08

**Authors:** L Bhatti, S Shah, H Khanlou

**Affiliations:** 1 AIDS Healthcare Foundation, Research, Beverly Hills, USA

## Background

Approximately 30% of HIV-infected patients are co-infected with hepatitis C (HCV). The current treatment standard of care for HCV, pegylated interferon and ribavirin (RBV), has demonstrated a sustained virologic response (SVR) in less than 50% of HCV/HIV co-infected patients, and only 17-35% in HCV genotype 1 patients. It has been previously shown that using a weight-based RBV dose results in favorable SVR rates. Prior studies suggest that HCV/HIV co-infected patients receiving a HAART regimen that included tenofovir (TDF) had higher SVR rates than those who received abacavir (ABC) in their nucleos(t)ide analogue (N(t)RTI) backbone.

## Purppose of the study

At our specialty clinic for the treatment of HCV/HIV co-infected patients, we re-examined the efficacy of HCV treatment in patients receiving either agent in their regimen with weight-based ribavirin doses.

## Methods

Patients with HIV/HCV co-infection (HCV genotype 1) met with a multidisciplinary team before therapy initiation for education and teaching. HCV treatment consisted of weekly injections of 180 mcg pegylated interferon subcutaneously and weight-based dosing of RBV (13mg/kg/day to maximum of 1200 mg/day). Treatment duration was 48 weeks with longer treatment in slow responders; side effects and adverse events were treated promptly. The HAART regimen consisted of a N(t)RTI backbone with either ABC or TDF and a protease inhibitor or non-NRTI. We retrospectively compared SVR rates in patients being treated with either ABC or TDF.

## Results

Thirty-four patients met the inclusion criteria. In an ITT analysis, 20 of 34 (59%) patients receiving HAART demonstrated SVR with no significant differences between races (p=.31). Among those twenty HAART patients with SVR, 9 patients were being treated with ABC and 11 were being treated with TDF (p=.13). The length of treatment between ABC and TDF treated groups did not differ significantly (49.6 and 49.5 weeks, p=.001). No significant difference in SVR rates was shown between the two groups.

## Conclusions

The rate of SVR in patients with HIV/HCV genotype 1 dosed with weight-based RBV was significantly higher than generally reported. There was no difference in SVR rates in HIV/HCV co-infected patients receiving ABC or TDF in their HAART regimen with weight-based RBV. These results may give providers flexibility in their selection of N(t)RTI backbone while receiving treatment for HCV.

